# Individual variations in nitric oxide synthase‐dependent sweating in young and older males during exercise in the heat: role of aerobic power

**DOI:** 10.14814/phy2.13208

**Published:** 2017-03-22

**Authors:** Tatsuro Amano, Naoto Fujii, Jeffrey C. Louie, Robert D. Meade, Glen P. Kenny

**Affiliations:** ^1^ Laboratory for Exercise and Environmental PhysiologyFaculty of EducationNiigata UniversityNiigataJapan; ^2^ Human and Environmental Physiology Research UnitUniversity of OttawaOttawaCanada; ^3^ Institute of Health and Sport SciencesUniversity of TsukubaTsukuba CityJapan

**Keywords:** Aerobic power, aging, eccrine sweat glands, exercise training, nitric oxide

## Abstract

We evaluated the association between aerobic power (defined by peak oxygen consumption; *V*O_2peak_) and the contribution of nitric oxide synthase (NOS) to the sweating response in young and older individuals during exercise in the heat. Data from 44 young (24 ± 1 years) and 48 older (61 ± 2 years) males with mean VO_2peak_ of 47.8 ± 2.4 (range, 28.0–62.3) and 39.1 ± 2.3 (range, 26.4–55.7) mLO_2_ kg^−1^ min^−1^, respectively, were compiled from our prior studies. Participants performed two 15‐ to 30‐min bouts of exercise at a fixed rate of metabolic heat production of 400 or 500 W, each separated by 15–20 min recovery in the heat (35°C, relative humidity of 20%). Forearm sweat rate (ventilated capsule technique) was measured at two skin sites that were continuously and simultaneously administered with lactated Ringers solution (Control) or 10 mmol/L *N*^*G*^‐nitro‐_L_‐arginine methyl ester (_L_‐NAME, nonselective NOS inhibitor) via intradermal microdialysis. Sweat rate during the final 5 min of each exercise bout was lower with _L_‐NAME compared to the Control in both groups (all *P *<* *0.05). The magnitude of the attenuation in sweat rate induced by _L_‐NAME compared to the Control was not correlated with *V*O_2peak_ (all *P *≥* *0.46) while this attenuation was negatively correlated with the sweat rate at the Control in both groups and in both exercise bouts (all *P *<* *0.01, *R* ≤ −0.43). These results suggest that NOS‐dependent sweating is not associated with aerobic power *per se*, while it becomes evident in individuals who produce larger sweat rates during exercise irrespective of age.

## Introduction

In humans, the evaporation of sweat is the major avenue of heat loss during exercise and exposure to hot ambient environments. Recent studies have demonstrated that nitric oxide synthase (NOS) is an important modulator of sweat rate in young adults during exercise (Welch et al. 2009; Fujii et al. 2014, 2016; Louie et al. 2016a). For instance, Welch et al. (2009) reported a reduction in sweat rate during exercise in warm conditions in young adults during local NOS inhibition; a response which has been confirmed in subsequent studies (Fujii et al. 2014, 2016; Stapleton et al. 2014b; Louie et al. 2016a). On the other hand, the contribution of NOS to sweating during exercise is diminished in older relative to young adults (Stapleton et al. 2014b; Fujii et al. 2015, 2016), which parallels the age‐related reductions in the sweating response (Kenney and Munce 2003; Inoue et al. 2004; Larose et al. 2013; Stapleton et al. 2014a, 2015). Interestingly however, recent reports suggest that inter‐individual variation in NOS‐dependent sweating exists in both young (Fujii et al. 2016) and older (Fujii et al. 2015) adults. While many factors such as age (Kenney and Munce 2003; Inoue et al. 2004; Larose et al. 2013; Stapleton et al. 2014a, 2015) and aerobic power (Greenleaf et al. 1972; Buono and Sjoholm 1988; Yamazaki et al. 1994; Yoshida et al. 1997; Ichinose‐Kuwahara et al. 2010; Amano et al. 2013) could influence sweat rate during exercise, the mechanisms underlying the inter‐individual variation in NOS‐dependent sweating have not been fully investigated.

Aerobic power is an important modulator of sweat production such that both young (Greenleaf et al. 1972; Yamazaki et al. 1994; Yoshida et al. 1997; Ichinose‐Kuwahara et al. 2010; Amano et al. 2013) and older (Drinkwater et al. 1982; Havenith et al. 1995; Stapleton et al. 2015) adults with elevated peak oxygen consumption (*V*O_2peak_) demonstrate an increased sweating response during exercise‐induced heat stress, which may in part be associated with enhanced cholinergic responsiveness and or increase in sweat gland size (Sato and Sato 1983; Sato et al. 1990) relative to their less fit counterparts. It has also been reported that exercise training (and thus potentially elevations in aerobic power) improves vascular function through NOS‐related mechanisms in young (Boegli et al. 2003; Montero et al. 2014) and older adults (Taddei et al. 2000; Black et al. 2008; Seals et al. 2008; Montero et al. 2014). Altogether these findings suggest that aerobic power may be an important modulator of NOS‐dependent sweating and may therefore contribute to the inter‐individual variation in NOS‐dependent sweating in both young and older individuals.

The purpose of this study was to investigate the association between aerobic power, as defined by *V*O_2peak_, and the contribution of NOS to sweating in young and older adults during exercise in the heat. We hypothesized that NOS‐dependent sweating would be associated with aerobic power in both young and older individuals. Data were compiled from previous studies conducted in our laboratory wherein the evaluation of NOS‐dependent sweating response during exercise in the heat was conducted using similar exercise protocols (Fujii et al. 2014, 2015; Stapleton et al. 2014b; Louie et al. 2016a).

## Materials and Methods

### Ethical approval

The included studies were approved by the University of Ottawa Health Sciences and Science Research Ethics Board and meet the guidelines set forth by the Declaration of Helsinki. Verbal and written informed consent was obtained from all volunteers prior to their participation.

### Participants

Data from 44 young and 48 older males were compiled for this report. The age, height, weight, body surface area and *V*O_2peak_ of participants in each group are presented in Table 1.

**Table 1:** Physical characteristics of young and older individuals

	Age (years)	Height (m)	Weight (kg)	Body surface area (m^2^)	Absolute *V*O_2peak_ (mLO_2_ min^−1^)	Relative *V*O_2peak_ (mLO_2_ kg^−1^ min^−1^)
Young adults						
Mean	24 (1)	1.77 (0.02)	78.5 (3.1)	1.95 (0.05)	3667 (165)	47.8 (2.4)
Highest	34	1.90	109.4	2.20	4577	62.3
Lowest	18	1.61	60.4	1.68	2394	28.0
Older adults						
Mean	61 (2)^a^	1.75 (0.02)	77.9 (3.0)	1.91 (0.04)	2941 (167)^a^	39.1 (2.3)^a^
Highest	74	1.86	99.1	2.21	4450	55.7
Lowest	51	1.65	57.8	1.63	1911	26.4

Values given are the means (95% confidence interval). *V*O_2peak_, peak oxygen consumption.

a Significant differences between groups (*P < *0.001).

### Experimental protocol

The experimental protocols employed have been reported elsewhere (Fujii et al. 2014, 2015; Stapleton et al. 2014b; Louie et al. 2016a) but are briefly described below.

### Preliminary testing session

Participants were required to undergo a preliminary testing session. During this time, their anthropometric and *V*O_2peak_ data were collected for screening purposes. Participants were asked to refrain from consuming food (≥2 h prior to the session), alcohol, caffeine, over‐the‐counter and/or prescriptions medications (including supplements such as vitamins and minerals) (≥24 h) and performing high‐intensity exercise (≥12 h) prior to the start of the experimental session. Body height and mass were determined using a stadiometer (Detecto, model 2391, Webb City, MO) and digital high‐performance weighing terminal (model CBU150X, Mettler Toledo Inc., Mississauga, ON, Canada), respectively, and these measurements were subsequently used to determine body surface area (Du Bois and Du Bois 1989). The hydrostatic weighing technique was utilized to determine body density, from which body composition was estimated (Siri 1956). We assessed *V*O_2peak_ using an incremental exercise protocol until exhaustion on a semirecumbent cycle ergometer (Corival Recumbent, Lode, Groningen, Netherlands). Expired air was continuously assessed during the exercise using an automated indirect calorimetry system (MCD Medgraphics Ultima Series, MGC Diagnostics, MN) and *V*O_2peak_ was taken as the greatest average oxygen uptake over a period of 30 sec. For the older adults (i.e., ≥55 years), a 12‐lead ECG was monitored throughout the maximal exercise test by a qualified technician to detect any abnormalities in heart activity. If abnormalities were detected, participants were excluded from the study and referred to their physician; however, no abnormalities were detected in the participants screened.

### Experimental session

On a separate day (≥48 h from the preliminary testing session), participants underwent the experimental testing session. Participants were instructed to follow the same pretrial instructions as the preliminary session prior to arriving to the laboratory on this day. Additionally, participants were instructed to adequately hydrate prior to the experimental testing session by consuming ≥500 mL of water the night prior and roughly 2 h before arriving to the laboratory. Urine samples were collected to assess urine specific gravity (Sawka et al. 2007) to confirm hydration status, for which the results were reported elsewhere (<1.020) (Fujii et al. 2014, 2015; Stapleton et al. 2014b; Meade et al. 2015; Louie et al. 2016a). While the participant rested in a thermoneutral ambient condition, four intradermal microdialysis fibers (30 kDa cutoff; MD2000, Bioanalytical Systems, West Lafayette, IN) were positioned under aseptic conditions in the dermal layer of skin on the dorsal side of the left forearm. This was accomplished using a 25‐gauge needle introduced into the nonanesthetized skin which travelled ~2.5 cm before exiting. Following the needle placement, the microdialysis fiber was threaded through the needle's lumen. By carefully withdrawing the needle, the 10 mm semipermeable membrane of the microdialysis fiber was situated in the forearm skin. The fiber was then secured in place to the skin using surgical tape. This process was repeated for the placement of up to four fibers [done for the assessment of different mechanisms as part of other studies (Fujii et al. 2014, 2015; Meade et al. 2015; Louie et al. 2016a)], each separated by 2–4 cm.

After placement of the intradermal microdialysis fibers, participants entered a temperature controlled chamber (Can‐Trol Environmental Systems Limited, Markham, ON, Canada) regulated to 35°C and 20% relative humidity where they rested on a semirecumbent cycle ergometer (Corival; Lode BV) for a minimum of 75 min. During this time, the microdialysis probes were perfused at a rate of 2–4 *μ*L min^−1^ in a counter‐balanced manner to receive (1) lactated Ringer solution (Baxter, Deerfield, IL) or (2) 10 mmol/L *N* ^*G*^‐nitro‐_L_‐arginine methyl ester (_L_‐NAME, Sigma‐Aldrich, St. Louis, MO). The concentration of _L_‐NAME was employed based on previous studies in which intradermal microdialysis was performed in human skin (Minson et al. 2001; Holowatz et al. 2005; Kellogg et al. 2005; Shibasaki et al. 2007; Wong 2013). The other two sites were perfused with other drugs, the results for which were published elsewhere (Fujii et al. 2014, 2015; Louie et al. 2016a). Each drug was perfused for a minimum of 60 min prior to the experimental protocol. A minimum of 90 min had elapsed following the insertion of the fiber and the start of the experiment. This time period has been shown to be sufficient to allow the insertion‐related trauma to subside (Hodges et al. 2009).

After 10 min of baseline resting data collection, participants performed two successive 15–30 min bouts of semirecumbent cycling at a fixed rate of metabolic heat production of either 400 or 500 W, with 15–20 min of recovery between exercise bouts. A fixed rate of heat production (heat load) was employed to ensure a similar thermal drive and therefore stimulus for sweating between individuals (Gagnon et al. 2013).

### Measurements

Heart rate was measured using a heart rate monitor (RS400, Polar Electro Oy, Kempele, Finland). Esophageal temperature was measured with a general purpose thermocouple (Mallinckrodt Medical, St Louis, MO), inserted through the nares to a depth of 40 cm past the entrance of the nostril. Skin temperature was measured using thermocouples (Concept Engineering, Old Saybrook, CT) attached to the skin with surgical tape. Mean skin temperature was calculated using four (Stapleton et al. 2014b) or six (Fujii et al. 2014, 2015; Meade et al. 2015; Louie et al. 2016a) skin sites. All temperature data were collected using a data acquisition module at a sampling rate of 15 sec and simultaneously displayed and recorded in spreadsheet format on a personal computer with LabVIEW software (Version 7.0, National Instruments, Austin, TX).

Sweat rate was measured continuously using the ventilated capsule technique. The capsule was affixed to the skin using adhesive rings and topical skin glue (Collodion HV, Mavidon Medical products, Lake Worth, FL). Dry compressed air was passed through each capsule and the moisture content of the effluent air was measured using a capacitance hygrometer (HMT 333, Vaisala, Helsinki, Finland). Long vinyl tubes were used to connect the gas tank (inflow) and hygrometers (outflow) to the sweat capsule (all located in the thermal chamber) to ensure that circulating gas temperatures were equilibrated to near room temperature (35°C). Local sweat rate was calculated every 5 sec using the difference in water content between the influent and effluent air, multiplied by flow rate, and normalized for the skin surface area under the capsule (expressed as mg min^−1^ cm^−2^).

### Data and statistical analyses

To investigate the validity of compiling data sets from intermittent exercise protocols of different duration and intensity, we compared the magnitude of the reduction in sweat rate between the Control and _L_‐NAME skin sites during the final 5 min of first and second exercise bouts between different exercise durations (15 or 30 min) and intensities (400 or 500W). Irrespective of the differences in exercise duration and intensity, we observed a similar reduction of sweat rate at the _L_‐NAME site relative to the Control in all comparisons for both young and older adults (all *P *>* *0.33). Given this observation, we compiled the data set for the analysis in this study.

Baseline variables were averaged over the 10 min prior to the start of the first exercise bout. The values for each intermittent exercise (exercise 1 and 2) and recovery (recovery 1 and 2) bout were obtained by averaging the final 5 min of each period. The difference in sweat rate between the Control and _L_‐NAME‐treated skin sites was calculated during exercise. In both the young and older groups, individual responses for sweat rate at the Control and _L_‐NAME sites as well as the difference in sweat rate between the Control and _L_‐NAME were plotted against *V*O_2peak_. In addition, the individual responses of sweat rate at the _L_‐NAME and the difference in sweat rate between the Control and _L_‐NAME during the first and second exercise bouts were plotted against sweat rate at the Control for each of the young and older participants.

A two‐way ANOVA was used for the comparisons of physiological measurements between young and older subjects and between treatment skin sites for sweat rate during exercise. The ANOVAs were performed with the factors of exercise time (five levels: baseline, exercise 1, recovery 1, exercise 2, and recovery 2) and treatment site (two levels: Control and _L_‐NAME) or age (two levels: young and older). Post hoc analyses were performed using Bonferroni's test. Linear regression analysis was performed to determine the relationship between dependent (sweat rate at the Control and _L_‐NAME sites as well as the difference in sweat rate between the Control and _L_‐NAME sites) and independent (*V*O_2peak_ and sweat rate at the Control site) factors. Data are presented as means ± 95% confidence interval (1.96 ×  standard error of the mean), and statistical significance was set at *P *<* *0.05. All statistical analyses were performed using GraphPad Prism (version 6.0, GraphPad software, La Jolla, CA).

## Results

The physiological responses measured during the intermittent exercise bout are presented in Table 2. Heart rate during exercise was greater in the young relative to older males (*P *<* *0.05) while it was similar between the groups during the recovery periods (*P *>* *0.05, Table 2). Esophageal and mean skin temperatures during exercise and recovery periods were not different between the groups (*P *>* *0.05, Table 2).

**Table 2:** Physiological variables during the experimental protocol

		Baseline	Exercise 1	Recovery 1	Exercise 2	Recovery 2
HR (beats min^−1^)	Y	73 (3)	127 (7)^b^	84 (4)	133 (7)^b^	88 (4)
	O	65 (3)	111 (6)	77 (5)	118 (7)	81 (5)
T_es_ (°C)	Y	37.05 (0.07)	37.60 (0.11)	37.33 (0.08)	37.83 (0.12)	37.48 (0.09)
	O	36.95 (0.07)	37.62 (0.10)	37.28 (0.07)	37.86 (0.10)	37.39 (0.07)
T_sk_ (°C)	Y	34.91 (0.13)	35.53 (0.18)	35.19 (0.21)	35.66 (0.17)	35.26 (0.19)
	O	34.81 (0.15)	35.30 (0.16)	35.02 (0.15)	35.37 (0.18)	35.01 (0.16)
Sweat rate at the Control site (mg min^−1^ cm^−2^)	Y	0.20 (0.03)	0.93 (0.13)	0.33 (0.08)	0.96 (0.14)	0.28 (0.04)
	O	0.18 (0.03)	0.84 (0.11)	0.37 (0.06)	0.85 (0.11)	0.29 (0.04)
Sweat rate at the _L_‐NAME site (mg min^−1^ cm^−2^)	Y	0.19 (0.03)	0.86 (0.12)^a^	0.31 (0.07)	0.89 (0.12)^a^	0.27 (0.04)
	O	0.17 (0.03)	0.79 (0.10)^a^	0.36 (0.06)	0.79 (0.11)^a^	0.28 (0.04)

Values given are the means (95% confidence interval). Y, young; O, older; HR, heat rate; T_es_, esophageal temperature; T_sk_, mean skin temperature.

a Significantly different from the Control site in each age group (*P < *0.05).

b Y versus O (*P < *0.05).

**Figure 1:**
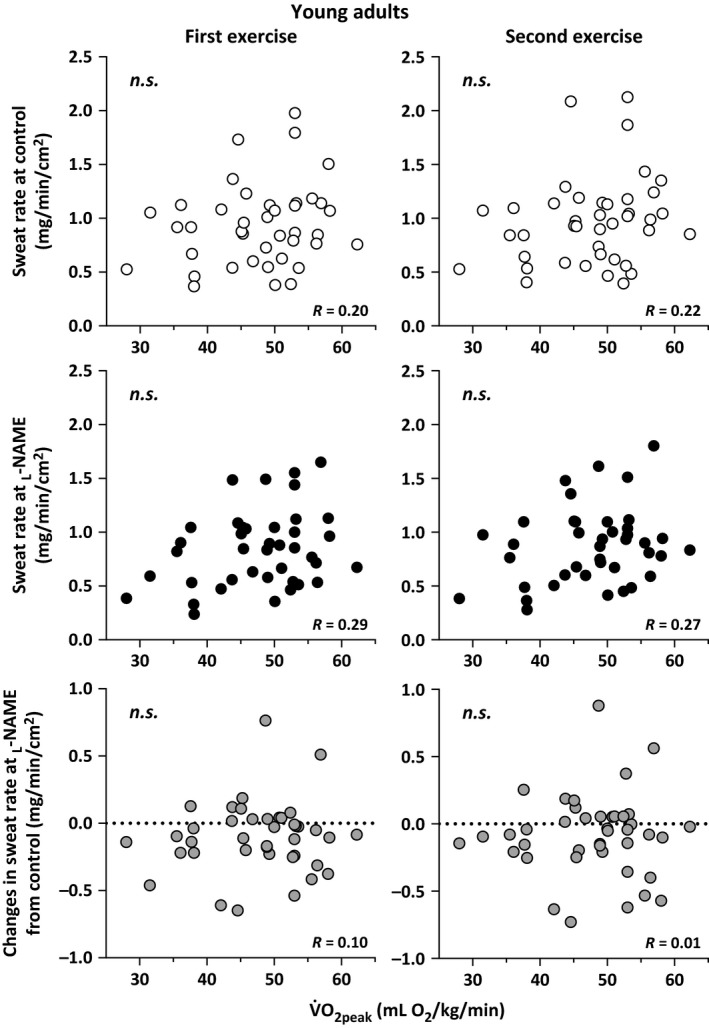
Individual variations in sweat rate at the Control and _L_‐NAME treated sites, and the change in sweat rate at the _L_‐NAME site relative to Control during the first and second bouts of intermittent exercise plotted against peak oxygen consumption (*V*O_2peak_) in young adults. n.s., not significantly different.

**Figure 2:**
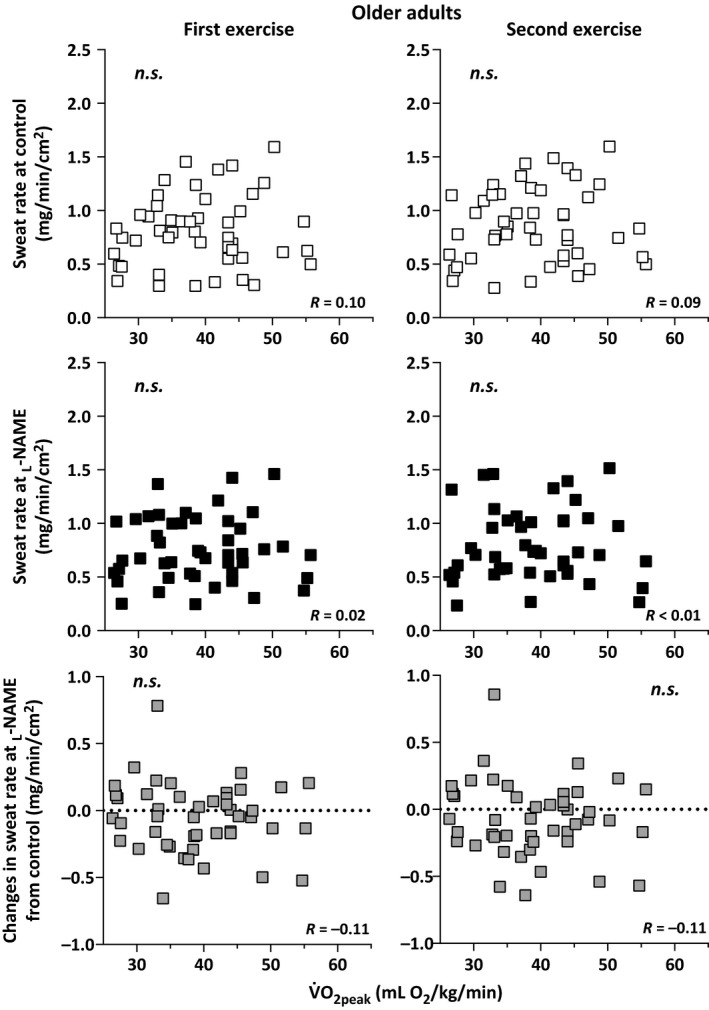
Individual variations in sweat rate at the Control and _L_‐NAME treated sites, and the change in sweat rate at the _L_‐NAME site relative to Control during the first and second bouts of intermittent exercise plotted against peak oxygen consumption (*V*O_2peak_) in older adults. n.s., not significantly different.

**Figure 3:**
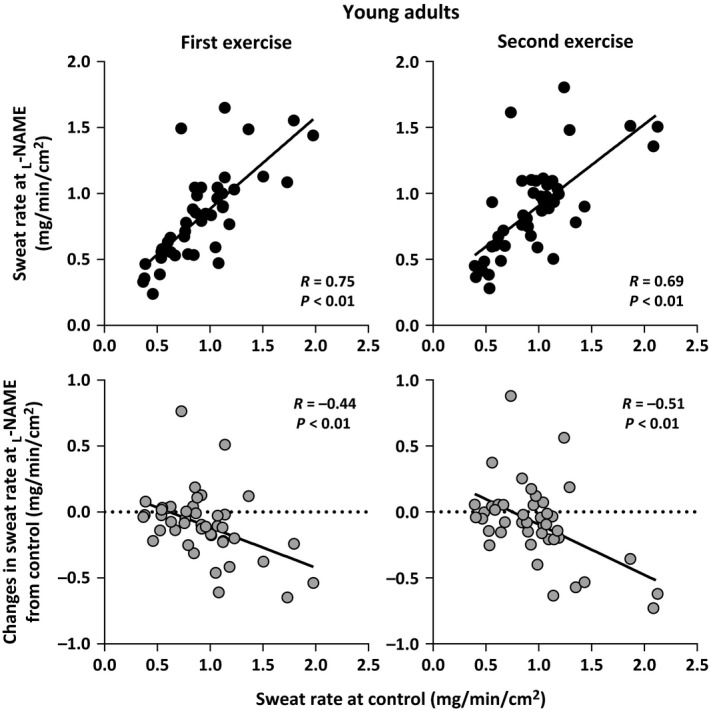
Individual variations in sweat rate at the _L_‐NAME treated sites and the change in sweat rate at the _L_‐NAME site relative to Control during the first and second bouts of intermittent exercise plotted against sweat rate at Control in young adults.

**Figure 4:**
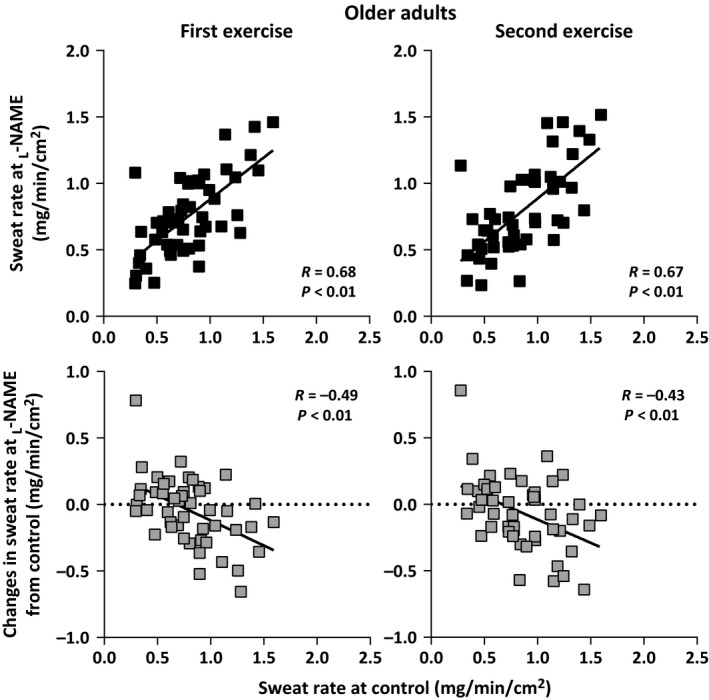
Individual variations in sweat rate at the _L_‐NAME treated sites and the change in the sweat rate at the _L_‐NAME site relative to Control during the first and second bouts of intermittent exercise plotted against sweat rate at Control in older adults.

Sweat rate at the Control and _L_‐NAME sites did not differ between the young and older adults during the first and second exercise/recovery bouts (all *P *>* *0.05, Table 2). Furthermore, in both age groups, sweat rate at the _L_‐NAME site was lower than that at the Control site during both exercise bouts (all *P *<* *0.05), but did not differ during the recovery periods (all *P *>* *0.05, Table 2). Sweat rate at the Control and _L_‐NAME sites as well as the difference in sweat rate between the Control and _L_‐NAME were not correlated with *V*O_2peak_ during both exercise bouts in either the young (all *P *>* *0.05; Fig. 1) or older (all *P *>* *0.05; Fig. 2) adults. In contrast, sweat rate at the _L_‐NAME (*R* = 0.67 to 0.75) was positively correlated with sweat rate at the Control site during both exercise bouts in the young (all *P *<* *0.01; Fig. 3) and older (all *P *<* *0.01; Fig. 4) adults. Finally, sweat rate achieved at the Control site and the difference in sweat rate between the Control and _L_‐NAME sites were negatively correlated during both exercise bouts in both young (all *P *<* *0.01; Fig. 3) and older (all *P *<* *0.01; Fig. 4) adults (*R* = −0.51 to −0.43).

## Discussion

To the best of our knowledge, this is the first study to evaluate the association between aerobic power and the contribution of NOS to the sweating response during exercise in the heat. Contrary to our hypothesis, the contribution of NOS to the sweating response (i.e., NOS‐dependent sweating) during exercise was not correlated with aerobic power (defined as *V*O_2peak_), in both the young and older adults. However, we showed that NOS‐dependent sweating was associated with the magnitude of sweating measured at the Control site during exercise and this response was consistent in both young and older adults. These results suggest that NOS‐dependent sweating would be most evident in individuals with elevated sweat outputs while aerobic power *per se* does not affect NOS‐dependent sweat production during exercise in the heat.

### Young adults

Previous studies have demonstrated that NOS is an important modulator of sweat rate in young adults during exercise (Welch et al. 2009; Fujii et al. 2014, 2016; Stapleton et al. 2014b; Louie et al. 2016a). Similar to these previous studies, which were conducted in a smaller number of subjects (10–12 young adults), we confirmed a reduction of sweat rate at the _L_‐NAME site relative to the Control site in this larger cohort (Table 2). In this study, we show that NOS‐dependent sweating was not correlated with aerobic power during exercise performed at a moderate fixed rate of metabolic heat production in the heat (35°C) (Fig. 1). This suggests that aerobic power *per se* does not explain the individual variation in NOS‐dependent sweating. It has been suggested that improvements in arterial vascular function following exercise training could be explained by both functional (i.e., improved bioavailability of endothelium‐derived NO) and structural (i.e., arterial remodeling) adaptations (Tinken et al. 2008; Green 2009). Functional adaptations can be observed following short‐duration exercise training (i.e., 2 weeks) and are superseded by arterial remodeling that typically occurs with longer training programs (Tinken et al. 2008; Green 2009). Taken together these findings support the possibility that the contribution of NOS to sweating may be dependent on both the duration and frequency of physical activity performed by the individual (and therefore level of habitual activity). While it is generally accepted that aerobic power (*V*O_2peak_) is a strong indicator of an individual's training status, a high *V*O_2peak_ is not necessarily indicative of a person's level of habitual activity (Martino et al. 2002). Physical activity is associated with partial heat acclimation, which can increase an individual's sweating response (Nadel et al. 1974). Given that the level of background physical activity was not assessed in our participants, it is possible that differences in habitual activity but not aerobic power may influence the relative contribution of NOS to the sweating response in this study. To clarify the direct influence of habitual activity and training per se further studies employing both cross sectional (e.g., trained versus untrained) and longitudinal (short‐term exercise training) exercise models are required.

Noteworthy, we found that the reduction in sweat rate induced by NOS inhibition was negatively correlated with the magnitude of sweat production observed at the Control site during exercise (Fig. 3). This suggests that the NOS‐dependent mediation of sweat production would be most evident in individuals with a relatively higher sweat rate during exercise; a response which occurs independent of an individual's aerobic power. However, a linear regression of the difference in sweat rate between the Control and _L_‐NAME relative to the sweat rate at the Control yielded a coefficient of determination (*R* ^2^) of 0.19 and 0.26 for the first and second exercise bouts, respectively (Fig. 3). This suggests that only 19‐26% of the variation in NOS‐dependent sweating is explained by the level of sweating at the Control site in young adults. The precise mechanism(s) underpinning the variations in NOS‐dependent sweating, however, cannot be fully elucidated from this study. It has previously been suggested that individual variations in sweat rate are associated with sweat gland size and/or cholinergic sensitivity/responsiveness of the eccrine sweat glands (Sato and Sato 1983; Sato et al. 1990). Thus, it is plausible that the individual variation in NOS‐dependent sweating may be influenced by variations in sweat gland size and/or cholinergic mediation of eccrine sweating.

A correlation between *V*O_2peak_ and the magnitude of sweat production during exercise‐induced heat stress has been observed in young adults (Greenleaf et al. 1972; Yoshida et al. 1997). For example, Yoshida et al. (1997) reported that *V*O_2peak_ is positively correlated with total sweat loss during light intensity exercise (i.e., 40% *V*O_2peak_) in the heat. In the present study, however, we did not observe a significant correlation between sweat rate and *V*O_2peak_ (Fig. 1). This may be attributable to the fact that in this study exercise was performed at a fixed rate of metabolic heat production whereas a relative exercise intensity (i.e., % of *V*O_2peak_) yielding different metabolic heat loads, and therefore thermal drive for sweating, was used in previous studies (Greenleaf et al. 1972; Yoshida et al. 1997). Moreover, we employed a moderate fixed rate of heat production which may have influenced the response. Fitness related differences in sweating are most evident at heat loads ≥300 W, and therefore moderate or higher intensities of exercise heat load (Louie et al. 2016b).

### Older adults

The sweating response in our older adults was similar to that of their younger counterparts (Fig. 2 and 4). Namely, individual variations in NOS‐dependent sweat production in older adults were not related to aerobic power. They were however associated with the magnitude of the sweating observed at the Control site. It has recently been reported that older individuals (*n* = 12) who have an elevated sweating response demonstrated a more pronounced attenuation in sweat rate following the administration of _L_‐NAME during exercise in hot conditions (Fujii et al. 2015). We confirm a similar response in this study but in a much larger sample size (Fig. 2). Finally, in contrast to recent reports (Stapleton et al. 2014b; Fujii et al. 2015), we observed a marked attenuation in sweat rate during local nonselective NOS inhibition (via _L_‐NAME) in older adults. Likely, this discrepancy is due to the comparatively greater number of participants examined in this study.

### Perspectives and Significance

NOS‐dependent sweat production has been well documented since it was first reported by Lee and Mack (2006). Subsequently, a number of studies have provided new insights into the mechanisms governing this response including the role of NOS in modulating sweating during exercise in the heat (Welch et al. 2009; Fujii et al. 2014, 2016; Stapleton et al. 2014b; Louie et al. 2016a). The current study extends upon these studies by demonstrating that aerobic power is not an underlying factor contributing to the variability of NOS‐dependent sweating. Further research is required to delineate the mechanisms underpinning the individual variations observed in NOS‐dependent sweating and the potential influence of habitual activity and training status in both young and older populations.

While the magnitude of NOS‐dependent sweating was relatively small during exercise (Table 2), this should not undermine the important contribution of a NOS‐dependent individual variation of sweating. It has been reported that short‐term heat acclimation can induce improvements of local sweating response of as much as 18–50% (Buono et al. 2009; Lorenzo and Minson 2010). Thus, even a minor contribution of NOS to a heat‐induced adaptation of the sweating response may be important.

In conclusion, we show that NOS mediates sweating during moderate intensity exercise in the heat in young and older adults, however, this is not associated with the individual's aerobic power. Further, we demonstrated that NOS‐dependent sweating is most evident in individuals with a relatively higher sweating response during an exercise‐induced heat stress.

## Conflicts of Interest

None.
